# Keratotic papules on the thigh: underrecognized skin manifestations of dermatomyositis or Wong-type dermatomyositis?^☆^

**DOI:** 10.1016/j.abd.2021.03.018

**Published:** 2022-12-19

**Authors:** Miyuki Yamamoto, Toshiyuki Yamamoto

**Affiliations:** Department of Dermatology, Fukushima Medical University, Fukushima, Japan

Dear Editor,

A 53-year-old female, who was admitted to the Rheumatology Department of our hospital, complaining of muscle weakness and joint pain, was referred to us regarding the skin symptoms which appeared 4 months previously. Physical examination revealed forehead and nasolabial erythema, periungual erythema, scaly erythema on the left knee, keratotic erythematous patch on the dorsum of metacarpophalangeal joint (Gottron’s sign), and keratotic lesion on the radial aspect of the second fingers (mechanic’s hand). In addition, small reddish, keratotic papules and papular erythemas were disseminated on the lateral aspects of the right thigh (Fig. 1). Laboratory examination showed elevation of aspartate aminotransferase (180 U/L), alanine aminotransferase (100 U/L), lactate dehydrogenase (487 U/L), creatine kinase (3506 IU/L), aldolase (35.8 U/L), and myoglobin (1230 ng/mL). The antinuclear antibody was positive (1:1280, speckled). Serum antibodies against TIF-1γ (48.0 index; normal <32) and Mi-2 (>150 index; normal <53) were elevated, whereas both anti-Jo-1 and anti-MDA-5 antibodies were normal. Serum KL-6 level was normal, and no interstitial lung disease (ILD) was detected. Skin biopsy revealed a keratotic plug, epidermal atrophy, liquefaction of the basal layers, individual cell keratinization, and infiltration of inflammatory cells in the papillary dermis (Fig. 2). No internal malignancy was detected by a detailed examination. An electromyogram of the biceps muscle revealed a myogenic pattern. MRI showed high signal intensity in the upper arm and thigh muscles. She was successfully treated with methylprednisolone pulse therapy followed by oral prednisolone.

**Figure 1:**
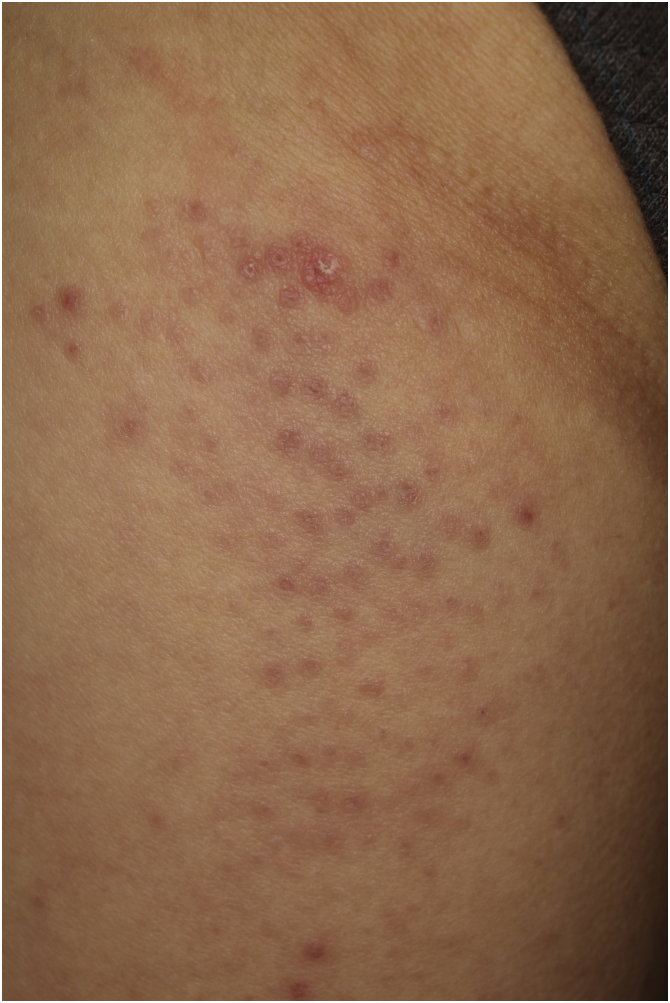
Clinical features of follicular papules on the thigh.

**Figure 2:**
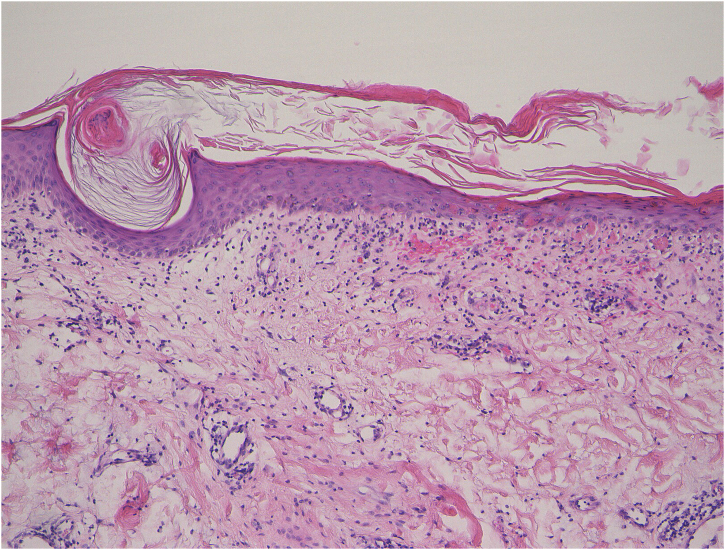
Histological features showing keratotic plug, epidermal atrophy, liquefaction of the basal layers, individual cell keratinization, and mononuclear cell infiltration in the upper dermis: (Hematoxylin & eosin 100 ×).

Wong-type dermatomyositis (DM) is characterized by keratotic erythematous follicular papules, which histopathologically show follicular hyperkeratosis with keratotic plugs filling dilated follicular infundibula.^1^ Whether this type is a distinct subtype of DM or not is still controversial. Our case presented various skin symptoms related to DM, and thus we think that keratotic papules on the thigh in the present case may better be considered as a rare manifestation of DM, rather than a Wong-type DM.

The association between Wong-type DM and either malignancy or ILD remains unclear. As far as we searched, 35 cases of Wong-type DM have been reported in the literature, which included cases with pityriasis rubra pilaris-like appearance, and cases with follicular keratotic papules on the buttock or extremities along with other various skin manifestations compatible with DM. Wong et al.^1^ reported that 52% of 23 patients (11 Wong-type and 12 typical DM) had complications of malignancy, but the frequency of either malignancy or ILD in Wong-type DM is uncertain. After excluding this report, we examined the 24 cases with Wong-type DM. Internal malignancy was observed in 3 cases among 17 cases with a description of malignancy (7 were unknown).^2^, ^3^, ^4^ And ILD was observed in only 1 case among 8 cases (16 were unknown). Our case did not have either ILD or internal malignancy; however, the patient is under careful follow-up because she had a positive anti-TIF-1γ antibody. The anti-TIF-1γ antibody is closely related to cancer-associated DM, and adult patients with this antibody are reported to have malignancy at a rate of 65%.^5^ Further studies are necessary to examine the relationship between myositis-specific antibodies and keratotic papular lesions.

## Financial support

None declared.

## Authors' contributions

Miyuki Yamamoto: Data collection, analysis, and interpretation; preparation and writing of the manuscript.

Toshiyuki Yamamoto: Approval of the final version of the manuscript.

## Conflicts of interest

None declared.
